# It's not all in your car: functional and structural correlates of exceptional driving skills in professional racers

**DOI:** 10.3389/fnhum.2014.00888

**Published:** 2014-11-11

**Authors:** Giulio Bernardi, Luca Cecchetti, Giacomo Handjaras, Lorenzo Sani, Anna Gaglianese, Riccardo Ceccarelli, Ferdinando Franzoni, Fabio Galetta, Gino Santoro, Rainer Goebel, Emiliano Ricciardi, Pietro Pietrini

**Affiliations:** ^1^Laboratory of Clinical Biochemistry and Molecular Biology, Department of Experimental Pathology, Medical Biotechnologies, Infectivology and Epidemiology, University of PisaPisa, Italy; ^2^Clinical Psychology Branch, University of Pisa, Azienda Ospedaliero Universitaria Pisana, Santa ChiaraPisa, Italy; ^3^MRI Laboratory, Fondazione Regione Toscana/Consiglio Nazionale delle Ricerche ‘G.Monasterio,’Pisa, Italy; ^4^Formula MedicineViareggio, Italy; ^5^Sport Medicine Unit, Department of Clinical and Sperimental Medicine, University of Pisa, Azienda Ospedaliero Universitaria Pisana, Santa ChiaraPisa, Italy; ^6^Maastricht Brain Imaging Center, Universiteit MaastrichtMaastricht, Netherlands

**Keywords:** inter-subject correlation, expertise, fMRI, functional connectivity, plasticity, voxel-based morphometry

## Abstract

Driving is a complex behavior that requires the integration of multiple cognitive functions. While many studies have investigated brain activity related to driving simulation under distinct conditions, little is known about the brain morphological and functional architecture in professional competitive driving, which requires exceptional motor and navigational skills. Here, 11 professional racing-car drivers and 11 “naïve” volunteers underwent both structural and functional brain magnetic resonance imaging (MRI) scans. Subjects were presented with short movies depicting a Formula One car racing in four different official circuits. Brain activity was assessed in terms of regional response, using an Inter-Subject Correlation (ISC) approach, and regional interactions by mean of functional connectivity. In addition, voxel-based morphometry (VBM) was used to identify specific structural differences between the two groups and potential interactions with functional differences detected by the ISC analysis. Relative to non-experienced drivers, professional drivers showed a more consistent recruitment of motor control and spatial navigation devoted areas, including premotor/motor cortex, striatum, anterior, and posterior cingulate cortex and retrosplenial cortex, precuneus, middle temporal cortex, and parahippocampus. Moreover, some of these brain regions, including the retrosplenial cortex, also had an increased gray matter density in professional car drivers. Furthermore, the retrosplenial cortex, which has been previously associated with the storage of observer-independent spatial maps, revealed a specific correlation with the individual driver's success in official competitions. These findings indicate that the brain functional and structural organization in highly trained racing-car drivers differs from that of subjects with an ordinary driving experience, suggesting that specific anatomo-functional changes may subtend the attainment of exceptional driving performance.

## Introduction

Throughout the centuries, first by using animals (i.e., horses) and then by developing motor vehicles and airplanes, humans have been able to reach speeds and accelerations tens of times higher than those that we would otherwise encounter by moving around with our own “body machine.” In this regard, high-speed driving can be considered as a “para-physiological condition,” in which the brain is required to process motion and motor information in a much faster and more demanding way. Interestingly, recent evidence collected in various highly skilled populations, including elite athletes, suggests that the expertise subtending exceptional driving abilities, as those shown by Formula racing-car professional drivers, may be associated with specific changes in the morphological (Gaser and Schlaug, 2003a,b; Draganski et al., 2004; Roberts et al., 2010; Wei et al., 2011; Di Paola et al., 2013) and functional (Jancke et al., 2000; Haslinger et al., 2004; Kelly and Garavan, 2005; Milton et al., 2007; Kim et al., 2008; Del Percio et al., 2009; Wei and Luo, 2010; Chang et al., 2011; Seo et al., 2012) architecture of the brain (reviewed in Jancke, 2009a,b; Yarrow et al., 2009; Nakata et al., 2010; Herholz and Zatorre, 2012; Chang, 2014). Different mechanisms have been proposed to explain structural modifications in these groups, including the formation of new neurons or glial cells, increases in cell size or spine density and axonal growth (May et al., 2007; Draganski and May, 2008; May, 2011). From a functional viewpoint, expertise-dependent changes are usually associated with a reduced recruitment of task-related cortical regions, accompanied by a strengthening of skill-relevant inter-regional connections, characteristics though to reflect an increased “*neural efficiency*” (Chein and Schneider, 2005; Brancucci, 2012; Bernardi et al., 2013; Patel et al., 2013).

As far as exceptional driving abilities are concerned, we recently showed that Formula racing-car professional drivers, as compared to naïve control subjects, present not only *quantitative*, but also *qualitative* distinctive brain functional correlates even during simple motor reaction and visuo-spatial tasks, in which naïve controls obtain similar behavioral results (Bernardi et al., 2013). Specifically, we observed that skilled car drivers are characterized by a reduced brain cortical activation and by reinforced connectivity measures between task-relevant areas. Moreover, during the same tasks, these individuals showed a higher signal temporal variability, a recently proposed marker of functional efficiency and regional information integration (Garrett et al., 2010; Leo et al., 2012; Ricciardi et al., 2013).

While the above results from our study indicate functional modifications in brain areas associated with motor control and visuo-spatial abilities, which are certainly required for the exceptional performance levels shown by these individuals, no research to date has investigated how these highly skilled brains process the complex flux of information related to actual driving behavior. Moreover, it is still unknown whether the specific expertise in driving racing-cars may be associated with distinctive structural brain substrates, and whether these changes may be correlated with the level of performance reached by the driver. To pursue these open questions, we took advantage of the fact that passive observation of video-clips depicting human behaviors do evoke a brain response that largely overlaps with the one observed during the actual execution of the same activities (Calvo-Merino et al., 2005; Gazzola and Keysers, 2009), and that this functional representation is dependent on the level of expertise achieved by the observer (Calvo-Merino et al., 2006; Cross et al., 2006). Thus, we used functional magnetic resonance imaging (fMRI) to compare patterns of brain response and regional interaction during an ecological passive driving task in which professional and naïve drivers watched, from the driver's subjective perspective, a Formula One car running on different official circuits. In particular, a continuous stimulation paradigm was used in place of the more classical block design, as it allowed for the simulation of a more “natural” driving condition, with no interruptions and no need for a priori assumptions regarding the specific events that modulated brain activity (Spiers and Maguire, 2007a). Moreover, functional MRI analysis was performed employing an *Inter-Subject Correlation* approach (ISC; Hasson et al., 2004; Pajula et al., 2012), which uses the activity timecourse of each area and compare it across subjects of a specific group to provide an index that is related to regional activity modulation by the stimulus (Hasson et al., 2010). Importantly, previous studies demonstrated that the ISC approach can be successfully used to study spontaneous brain response during naturalistic stimulation in a completely data driven fashion (Jaaskelainen et al., 2008; Hasson et al., 2009; Kauppi et al., 2010; Nummenmaa et al., 2012). Finally, high-resolution MRI anatomical brain images of professional and naïve drivers were obtained to compare gray matter density in cortical and subcortical brain structures, using voxel based morphometry (VBM; Ashburner and Friston, 2000).

We anticipated that during passive driving, expert racing-car drivers would show a functional plastic adaptation of regions involved in visuo-spatial navigation and motor control, when compared to untrained naïve drivers. In particular, we hypothesized that these brain areas would be characterized by a greater inter-subject and intra-subject synchronicity (i.e., coupling), potentially related to the acquisition of specific behavioral and functional motor repertoires. In addition, we predicted that these driving-related brain areas would also be characterized by expertise-dependent structural changes, as expressed by an increased regional gray matter density.

## Material and methods

### Subjects

Eleven professional (mean age ± SD = 24 ± 4 years) and 11 naïve (28 ± 4 years; *p* = n.s.) car drivers participated in the MRI study, which comprised the acquisition of brain anatomical data and two distinct functional studies, the passive driving paradigm reported here and the motor-reaction and visuo-spatial tasks previously reported (Bernardi et al., 2013). Professional car drivers were recruited from the pool assisted by the Formula Medicine® group (Viareggio, Italy). All car racers were actively participating in a professional racing tournament (as Formula One Championship, World Series, Formula 3, etc.) at the time of the study and had a minimum of 4 year expertise in amateur and professional racing. Naïve car drivers were recruited from the general population and had no history of practicing any sport at an amateur or professional level. All subjects were right-handed healthy males. Clinical examinations and laboratory testing, including a structural brain MRI scan exam, were performed to rule out history or presence of any relevant medical, neurological or psychiatric condition that could affect brain function and development. All subjects were free of medications and gave their written informed consent after the study procedures and risks involved had been explained. The study was conducted under a protocol approved by the University of Pisa Ethical Committee (protocol n. 1616/2003), and was developed in accordance with the Protocol of World Medical Association (2008). All participants retained the right to withdraw from the study at any moment.

### Image acquisition

Functional and structural brain images were acquired on a GE Signa 1.5 Tesla scanner (General Electric, Milwaukee, WI). For each subject we obtained a high-resolution T_1_-weighted spoiled gradient recall image (slice thickness = 1 mm, echo time = 3.8 ms, repetition time = 20 ms, flip angle = 15°, field of view = 220 mm, acquisition matrix = 220 × 220, 150 axial slices) both to provide detailed brain anatomy for functional data localization and for structural analyses based on voxel based morphometry measures.

Functional data were collected using the following parameters: repetition time = 2500 ms, number of axial-slices = 21, slice thickness = 5 mm, field of view = 240 mm, echo time = 40 ms, flip angle = 90°, image plane resolution = 128×128. Because of technical reasons, functional data from the passive driving paradigm were not available in one out of the 11 professional drivers and in 2 out of the 11 naïve drivers. While in the magnetic resonance scanner, participants were presented with four video-clips recorded by an on-board camera placed on a Formula One car running on different circuits: Spa-Francorchamps (Spa, Belgium), Magny-Cours Circuit (Nevers, France), Autodromo Enzo e Dino Ferrari (Imola, Italy) and Bahrain International Circuit (Sakhir, Bahrain). Visual stimuli were presented on a rear projection screen viewed through a mirror (visual field: 25° wide and 20° high). All four video-clips were presented in a single continuous sequence (with a 1 s black screen separating each clip from the following) overall lasting 340 s (136 volumes). A black screen was shown at the beginning of each functional time series for 15 s (6 volumes) that were subsequently discarded to allow for magnetic field stabilization. To maximize compliance and attention to the stimuli, before the fMRI scanning subjects were instructed to imagine themselves driving the racing-car.

### Functional data preprocessing

We used AFNI and SUMA software packages to analyse and display functional imaging data (http://afni.nimh.nih.gov/afni; Cox, 1996, 2012). All obtained functional volumes were coregistered (*3dvolreg*), temporally aligned (*3dTshift*), and spatially smoothed using a Gaussian kernel of FWHM 8 mm (*3dmerge*). Individual run data were scaled by calculating the mean intensity value for each voxel during the entire functional run, and by dividing the value within each voxel by this averaged baseline to estimate the percent signal change at each time point. Additional preprocessing steps included removal of other effects of no interest, specifically, head motion, and drifting effects, from all timeseries. Individual preprocessed functional data were registered to the Talairach and Tournoux Atlas coordinate system (Talairach and Tournoux, 1988), and resampled into 2 mm^3^ voxels. Brain activations were anatomically localized on the naive and professional group-averaged Talairach-transformed T_1_-weighted images, and visualized on normalized SUMA surface templates.

### Inter-subject correlation analysis

The exploration of brain functional responses during a natural viewing condition is not easily attainable using classical analysis approaches based on general linear model (GLM) (Spiers and Maguire, 2007a). Thus, to determine the regional brain activation during continuous passive driving, we used an Inter-Subject Correlation (ISC) analysis (Hasson et al., 2004; Pajula et al., 2012). The ISC approach is based on the assumption that some events included in naturalistic stimuli are able to evoke functionally selective, time-locked, brain response with high reproducibility across different subjects, and thus operates in a completely data-driven fashion (Hasson et al., 2010).

Pearson's coefficient was used to determine correlation between every pair of subjects within each group on a voxel by voxel basis (Hasson et al., 2004). Thus, as we included 10 professional and 9 naive drivers, we obtained a total of 45 and 36 correlation maps, respectively, that were then used to calculate the averaged correlation coefficient per voxel in each group. To define significant correlations in the obtained group maps we performed a fully non-parametric voxel-wise permutation test using ISC-toolbox (Kauppi et al., 2010). This program generated the permutation distribution by circularly shifting each subject's time series by random amount so that they were no longer aligned, and then calculated the new correlation values. The full permutation distribution was approximated with 100,000,000 realizations for each group. Correction for multiple comparisons was attained using false discovery rate (FDR) with independence or positive dependence assumption (Benjamini and Hochberg, 1995; Nichols and Hayasaka, 2003; Kauppi et al., 2010). The significance threshold was set at FDR corrected *p* < 0.001.

To better characterize ISC differences between professional and naïve drivers, we computed a contrast between the two groups using an approach similar to the one described in Cantlon and Li (2013). Specifically, individual activation maps were obtained for each subject by averaging the ISC-maps representing the voxel-to-voxel correlations with all other subjects of the same group. Then, a whole brain statistical comparison was carried out via unpaired *t*-test over individual Fisher-transformed r-maps (*p* < 0.01, FDR corrected; a minimum volume threshold of 30 voxels was also applied to filter out smaller clusters).

### Functional connectivity analysis

In order to reduce the probability of identifying spurious correlations, the timeseries extracted from a single voxel located in the lateral ventricles, the six motion correction parameters derived from the volume registration and the polynomial regressors accounting for baseline shifts and linear/quadratic/cubic drifts were mathematically removed (*3dSynthesize*) from the preprocessed voxel timecourse (Lund et al., 2006). In addition, timeseries were low-pass filtered (*3dFourier*) at 0.15 Hz to remove high frequency physiological artifacts including cardiac and respiratory pulsatility (Birn et al., 2006).

We divided each brain hemisphere into 45 cortical and subcortical regions using the Eickhoff-Zilles Atlas (Eickhoff et al., 2005). Regional mean timeseries were estimated for each individual by averaging the fMRI timecourses over all voxels in each of the 90 regions. For each subject an individual correlation matrix was obtained by computing the Pearson's correlation coefficient between each region of interest and all other considered regions of the brain. In order to determine significantly different correlations between the two examined groups, we converted correlation coefficients of each subject into z-scores using Fisher's z transformation and then performed an unpaired *t*-tests at each location of the correlation matrix (*p* < 0.05).

### Voxel-based morphometry

The estimation of structural changes in cortical and subcortical brain regions was performed using an optimized voxel-based morphometry (VBM) protocol (Ashburner and Friston, 2000; Good et al., 2001), carried out with FSL tools (Smith et al., 2004). One naïve driver was excluded from this analysis due to MRI artifacts which compromised the VBM measure in occipital and parietal brain areas, thus structural data from 11 professional (24 ± 4 years) and 10 naïve (28 ± 4 years) drivers were used. First, high resolution anatomical brain images were extracted using the Brain Extraction Tool (BET; Smith, 2002), corrected for radio-frequency (RF) pulse inhomogeneity and segmented into different tissue types (gray matter, white matter and cerebrospinal fluid) through the FMRIB's Automated Segmentation Tool (FAST; Zhang et al., 2001). The resulting gray matter images were registered to the 2 mm isotropic MNI-152 atlas (Fonov et al., 2009) by means of non-linear registration (FNIRT; Andersson et al., 2007) and were averaged, as well as flipped along the x-axis to create a symmetric, study-specific gray matter template. Afterward, the native gray matter images were non-linearly registered to this template and modulated to correct for local expansion or contraction due to the non-linear component of the spatial transformation (multiplying the gray matter by the Jacobian of the warp field; Fonov et al., 2009). The modulated gray matter maps were then smoothed using an isotropic Gaussian kernel with a sigma of 3 mm.

To investigate differences between professional and naïve drivers, a voxel-wise group comparison test was applied by using a permutation-based non-parametric approach (*n* = 5000 permutations to estimate the null distribution), while correcting for multiple comparison and adding age and overall brain volume as nuisance variables (*p* < 0.05, corrected with threshold-free cluster enhancement, TFCE; Smith and Nichols, 2009). Further, to determine how driving expertise is related to functional and structural brain modifications in professional drivers, we ran a correlation analysis between gray matter density and a “driving proficiency index,” computed as the number of podia achieved during the entire career divided by the number of races entered at the time of the study (data extracted from the online “driver database” [http://www.driverdb.com/]). The number of podia per race, ranging from 0 (worst performance) to 1 (best performance), could be reasonably considered as a success index, since it is highly correlated with the number of races won (*r* = 0.89) and because each race placement defines the overall championship score. To this purpose, the same VBM preprocessing workflow described above was applied, resulting in a gray matter template specific for the professional driver subjects solely (*n* = 11). Next, functional results were spatially transformed from the Talairach to the MNI space in order to carry out a logical conjunction (AND) between ISC (*p* < 0.01, FDR corrected) and VBM (*p* < 0.05, TFCE corrected) group comparison maps. Afterward, a voxel-wise linear regression between gray matter density and driving proficiency index was applied within the resulting mask. By doing this, we identified brain regions, within those structurally related to group differences, which showed a morphological reorganization associated with driving expertise, hence mediated by its functional role. Statistical significance level was estimated by means of the non-parametric permutation test previously described (*p* < 0.05, small volume TFCE corrected).

## Results

### Inter-subject correlation analysis

**Figure 1:**
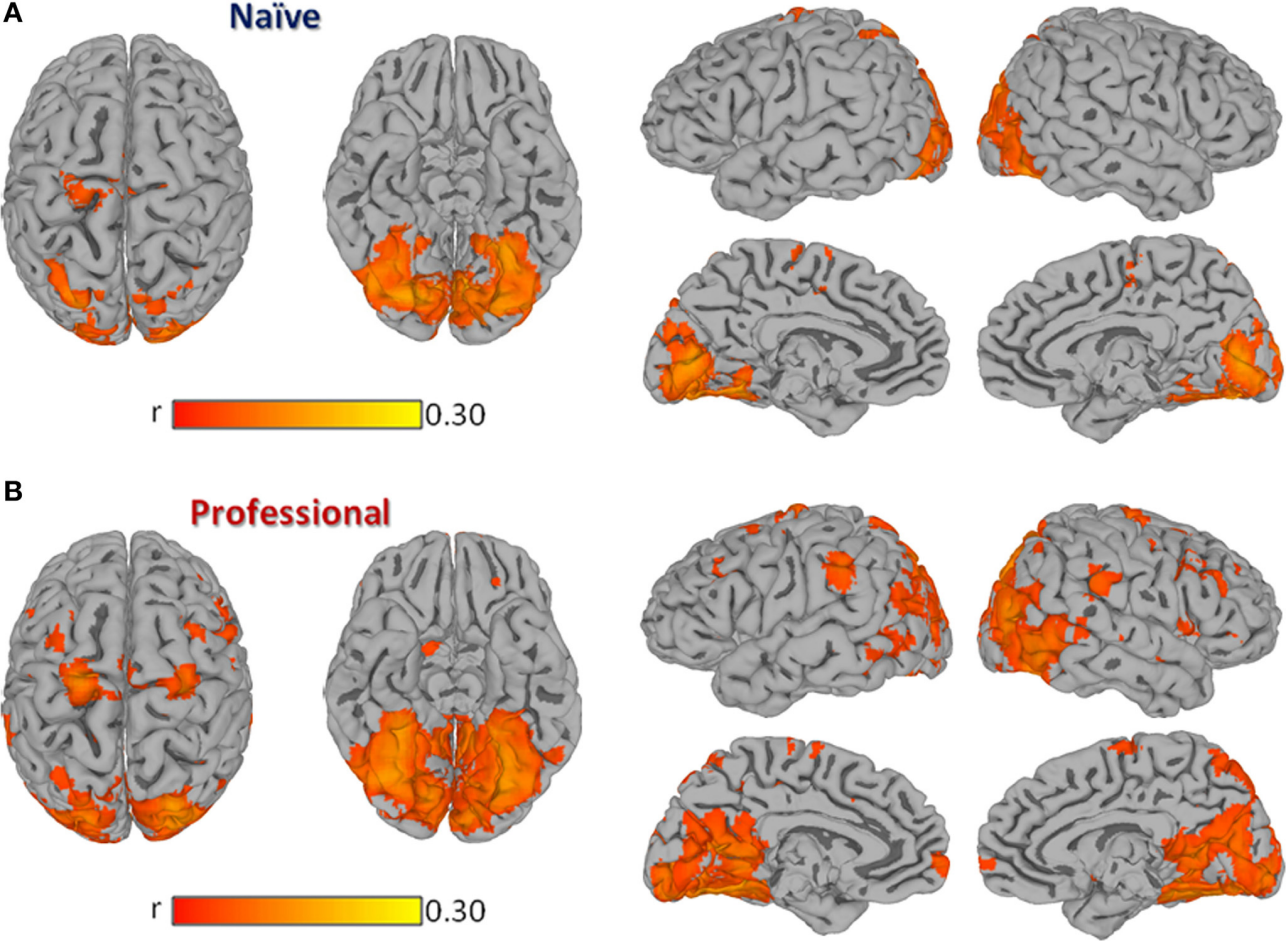
**Group Inter-Subject Correlation (ISC) maps obtained by averaging Pearson's correlation coefficients computed between each pair of subjects within the same group at each location**. Panel **(A)** shows the group-level ISC-map obtained in naïve drivers, while **(B)** shows results obtained in professional drivers. All results are FDR corrected *p* < 0.001.

In both professional and naïve drivers, passive driving significantly modulated regional activity in a set of cortical areas known to be involved in visual information processing, vigilance, attention, motor control, and more specifically, in driving behavior (Walter et al., 2001; Spiers and Maguire, 2007b). In fact, both groups showed a significant brain response in bilateral visual cortex (BA17, BA18, BA19), precuneus, cingulate, parahippocampus, superior parietal, medial frontal (BA6), right dorsolateral prefrontal (BA9) and left precentral cortex (Figure 1). However, professional drivers showed additional significant correlations in bilateral inferior parietal, inferior/middle temporal, medial/superior frontal, inferior frontal, left middle frontal, and right precentral cortex.

**Figure 2:**
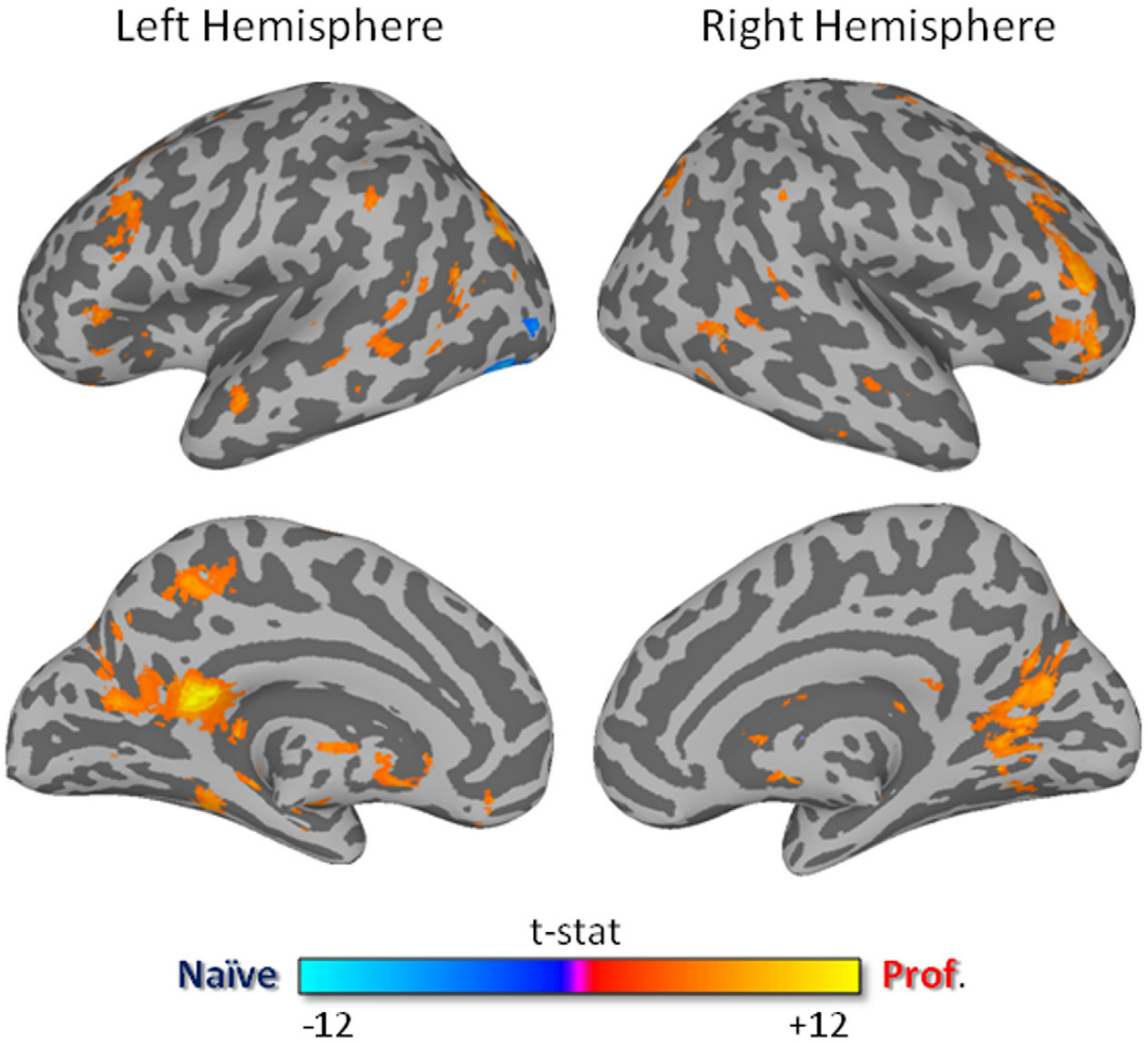
**Contrast between ISC-values of naïve and professional drivers**. The dark/light blue color indicates a higher correlation value in the naïve drivers, while red/yellow colors indicate a higher correlation in the professional drivers (*p* < 0.01, FDR corrected, minimum cluster size is set to 30 voxels).

The contrast carried out between the two groups revealed a significantly stronger correlation (*p* < 0.01, FDR corrected) in professional drivers, as compared to naïve drivers, in bilateral cingulate cortex and posterior cingulate, precuneus, parahippocampal, supramarginal, middle temporal, middle, and inferior frontal cortex, and caudate nucleus, left anterior cingulate cortex, medial frontal, and thalamus, right superior frontal and precentral cortex, lentiform nucleus, and cerebellum (Figure 2; Supplementary Table S1). On the other hand, naïve drivers showed stronger correlations only in the left middle occipital cortex.

### Functional connectivity analysis

**Figure 3:**
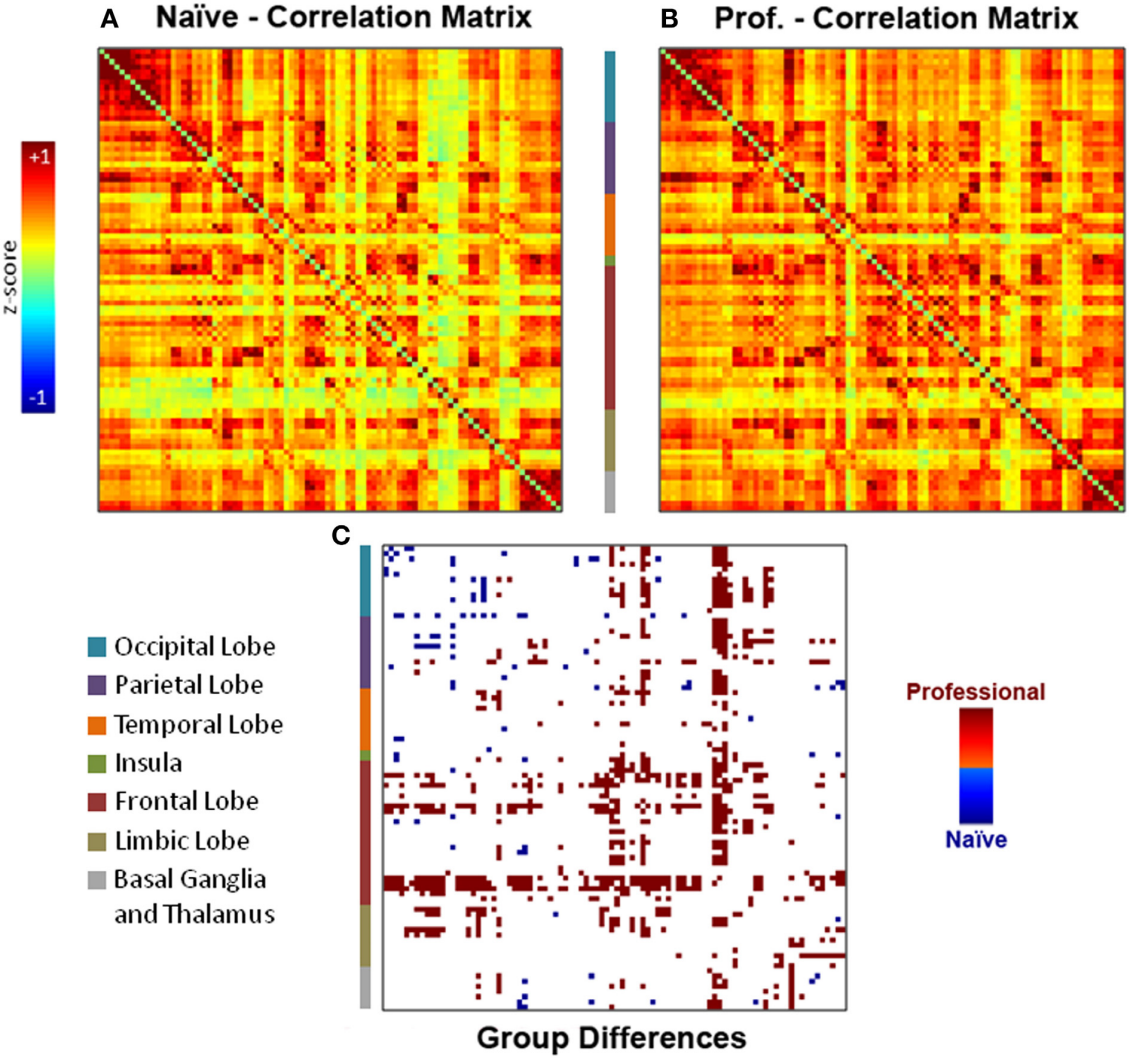
**Individual functional connectivity matrices obtained with the explorative approach were used to compute (A–B) group averaged matrices and (C) a comparison between professional and naïve drivers via unpaired *t*-test (*p* < 0.05)**. Results showed a number of reinforced correlations in professional as compared to naïve drivers, mostly in prefrontal cortex (orbitofrontal cortex, superior frontal gyrus), basal ganglia and cingulate cortex. On the other hand, naïve drivers showed fewer stronger correlations, mostly between areas belonging to occipital and parietal lobes.

Individual functional connectivity matrices obtained with an explorative approach were used to calculate averaged group maps (Figures 3A,B) and to compute a comparison between professional and naïve drivers (Figure 3C) via unpaired *t*-test (*p* < 0.05). Results showed a considerable number of reinforced correlations in professional as compared to naïve drivers, mostly involving prefrontal cortex, anterior and posterior cingulate and basal ganglia. In particular, areas that showed the greatest changes in inter-regional correlations included medial and orbitofrontal, superior frontal, cingulate, motor, and premotor cortical areas (Supplementary Table S2). On the other hand, naïve drivers showed fewer stronger correlations, mostly between areas belonging to striate and extrastriate visual regions and parietal cortex (Figure 3C). Importantly, the number of obtained results at α = 0.05 was significantly greater than what should be expected using Poisson distribution as a model (*p* = 2^*^10^−16^).

### Voxel-based morphometry

**Figure 4:**
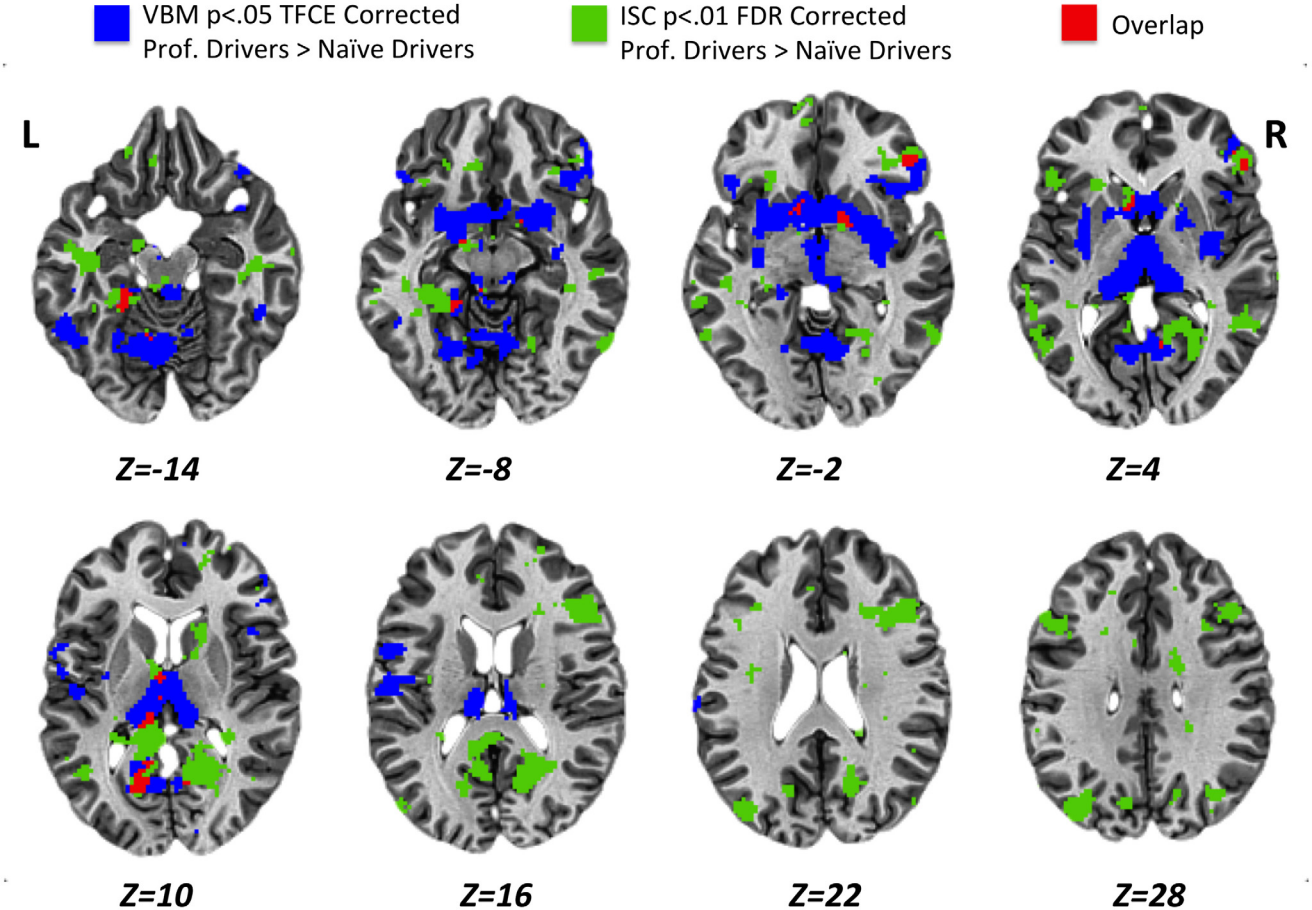
**Results obtained for VBM (*p* < 0.05 TFCE corrected) and ISC (*p* < 0.01 FDR corrected) analyses from the comparison between professional race car drivers and naïve subjects**. Blue voxels represent brain areas with a significantly higher gray matter density, while green voxels refer to regions that were significantly more activated in expert drivers, as compared to the naïve subjects, during the passive driving task. In red, overlap regions between VBM and ISC results.

Results obtained using the VBM analysis showed an increase in gray matter density across several brain regions for the professional racing-car drivers as compared to the naïve subjects (*p* < 0.05, TFCE corrected). These regions included the bilateral thalamus, lentiform and caudate nuclei, posterior cingulate and retrosplenial cortex (RSC, BA30), as well as the inferior temporal, the fusiform, the pars orbitalis and triangularis of the inferior frontal (BA45/BA47) and the precentral gyri (Supplementary Table S3). In addition, we found differences in the left lingual, left postcentral gyrus, left parahippocampal gyrus, and in the right medial frontal gyrus (BA10). No significant reductions (*p* < 0.05 TFCE corrected) in gray matter density were found in the professional race car drivers as compared to the control group (Figure 4, blue areas).

Statistical maps obtained from structural (VBM, Figure 4, blue areas) and functional (ISC, Figure 4, green areas) group comparison (professional > naïve drivers) revealed areas of overlap (Figure 4, red areas) for the bilateral posterior cingulate and retrosplenial cortex, left parahippocampal, left thalamus, left caudate nucleus, right lentiform nucleus, and right inferior frontal gyrus. No overlapping regions were found for the VBM and ISC analyses while contrasting naïve greater than expert drivers.

**Figure 5:**
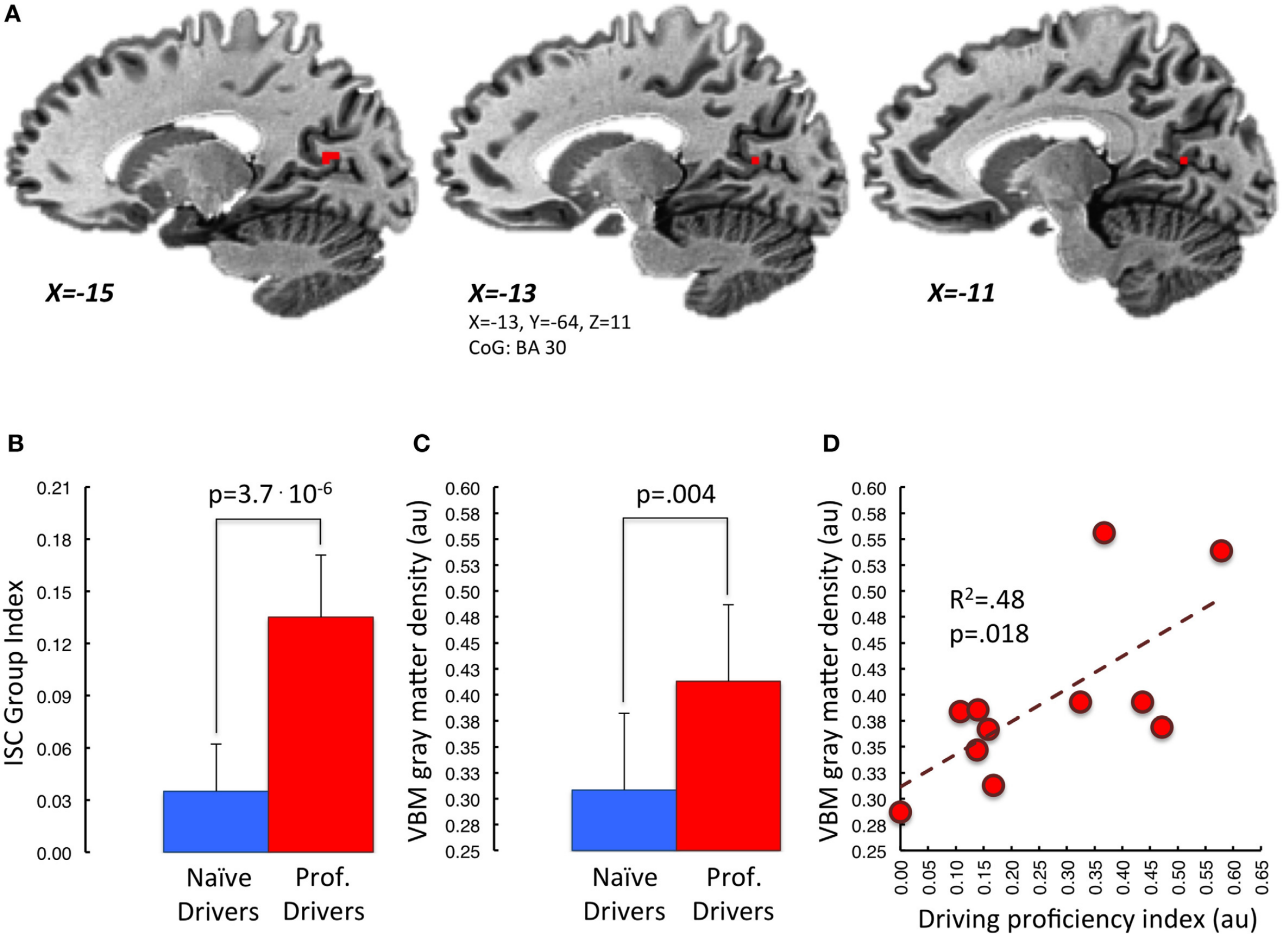
**Correlation between mean VBM-values in retrosplenial cortex and driving proficiency**. In **(A)** is represented in red the portion of BA30 lying in the left parieto-occipital sulcus. This area appears to be the one, among those showing functional and structural group differences, characterized by a significant correlation between gray matter density and “driving proficiency index” (*p* < 0.05 small volume TFCE corrected). Task-related Inter-Subject Correlation values and gray matter density measures of both groups extracted from this region of interest are represented in **(B,C)** respectively. Panel **(D)** depicts the correlation between cortical gray matter density and the performance level achieved in the professional drivers group (red dots). [au]: arbitrary units.

Further, the correlation analysis between gray matter density and driving proficiency index (podia divided by number of races), carried out within the previously described overlapping areas, revealed a significant positive correlation in the left retrosplenial cortex, BA30 (*p* < 0.05, small volume TFCE corrected; Figure 5). Noteworthy, using the same statistical threshold, we did not find any brain area showing a negative correlation between gray matter density and driving proficiency index.

## Discussion

In the present work, we used functional and structural MRI analyses to determine whether the exceptional levels of driving performance achieved by professional racing-car drivers after years of training and competitions are associated with distinctive brain functional and structural correlates as compared to untrained naïve drivers. Notably, the use of both structural and functional approaches in a single framework allowed us to look not only for specific differences between the two groups under each aspect, but also for possible interactions between functional and anatomical changes. To this aim, first, we compared patterns of brain activity and regional connectivity during a passive driving task in the two groups. As expected, the comparison of the two experimental groups revealed a more consistent functional recruitment of driving-related brain regions in professional than in naïve drivers. Moreover, a VBM analysis revealed that some of these areas also present an increased gray matter density, likely reflecting an association between the functional and structural plastic brain modifications that support top performance racing skills.

### Distinctive brain response modulation during passive driving in expert and naïve drivers

In both professional and naïve car drivers, passive driving was associated with the recruitment of various cortical and subcortical areas already identified by previous investigations aimed at defining the functional correlates of driving (Walter et al., 2001; Calhoun et al., 2002; Graydon et al., 2004; Horikawa et al., 2005; Jeong et al., 2006; Spiers and Maguire, 2007b; Mader et al., 2009; Calhoun and Pearlson, 2012). These regions included bilateral areas devoted to visual processing (striate and extrastriate cortex), superior and inferior parietal cortex, right dorsolateral prefrontal cortex and left sensorimotor cortex. However, as predicted, professional and naïve drivers had substantially different patterns of brain activity. In fact, while untrained naïve subjects showed a consistent modulation of brain response mostly limited to visual brain areas, professional drivers were characterized by greater BOLD signal synchronization in a number of additional cortical regions, including bilateral cingulate cortex, parahippocampus, precuneus, motor/premotor areas, dorsolateral prefrontal cortex, and middle temporal cortex. Most of these areas have been previously related to different aspects of driving behavior, including vigilance, visuo-spatial monitoring, and navigation, action preparation and motor control (Calhoun et al., 2002; Spiers and Maguire, 2007b).

Moreover, the exploratory connectivity analysis revealed significant differences in task-related regional interactions between the two experimental groups. In fact, professional drivers showed higher correlation measures, as compared to naïve drivers, in a number of brain regions, including prefrontal areas, anterior, and posterior cingulate cortex and basal ganglia. In particular, some of these regions, including the orbitofrontal, dorsomedial prefrontal, superior prefrontal, motor, and premotor cortical areas, were characterized by a high number of stronger functional couplings. On the other hand, naïve drivers were characterized by higher correlation measures in regions devoted to visual and spatial information processing. These findings are consistent with and reinforce the results obtained using the ISC analysis, indicating a stronger functional coupling between cortical areas involved in motor planning (e.g., premotor cortex, basal ganglia; Monchi et al., 2006; Spiers and Maguire, 2007b), motor control (e.g., basal ganglia, primary motor cortex; Monchi et al., 2006) and decision making (e.g., cingulate cortex, prefrontal cortex; Cohen et al., 2005; Rushworth et al., 2007), in the professional drivers as compared to the naïve individuals. Interestingly, the basal ganglia have been demonstrated to be fundamental in learning a new motor behavior and in retrieving its representation after the skill has become fully automatized (Doyon et al., 2009). Thus, our results show that, in racing-car drivers, cortical, and subcortical areas were actually exchanging information and working synergically at a significantly higher degree during the passive driving task, potentially retrieving, and representing information related to the acquired driving skills.

Overall, our results are consistent with previous findings in other expert groups, such as professional dancers, indicating that during passive viewing tasks brain response is strongly dependent on the expertise of the observer in the depicted activities (Calvo-Merino et al., 2005, 2006; Kim et al., 2011). For instance, a stronger response in various brain areas has been shown in expert archers as compared to untrained individuals, while they observed short movies depicting movements that only the first group had been trained to perform (Kim et al., 2011). Similar findings have been described using motor imagery paradigms, for example in skilled divers required to imagine movements specifically related to their sport activity (Wei and Luo, 2010). In line with these works, our results indicate that a specific motor expertise is required to obtain an actual motor representation, rather than simply a mere visual motor representation (Calvo-Merino et al., 2006). As a matter of fact, although both professional and naïve drivers may share the common knowledge necessary for ordinary road driving, driving a racing-car implies a number of additional skills, from the use of different controls to the management of braking and rapid accelerations. Indeed, the lack of a direct experience in driving racing-cars probably prevented the control group from attaining an actual motor representation, relegating the brain functional response to visual areas. Put it in a simpler way, naïve individuals simply watched the race, while professional drivers imagined themselves to race.

### Brain functional modifications are associated with specific structural changes

Recent evidence indicates that brain modifications induced by expertise acquisition are not just limited to the functional aspects, but are instead frequently associated with relevant anatomical adaptations (Draganski and May, 2008; May, 2011). Interestingly, a study performed by Sagi et al. (2012) demonstrated that just 2 h of practice with a driving simulator are sufficient to induce structural changes detectable using diffusion tensor imaging (DTI; Assaf and Pasternak, 2008). In particular, while the functional brain response was not investigated in their work, most of observed modifications involved areas previously associated with driving behavior and spatial navigation (Sagi et al., 2012). Given these premises, one could expect to find distinctive structural correlates in individuals exposed to many years of specific trainings and extreme competitive conditions, such as those present in high-speed car-racing championships.

As a matter of fact, by comparing brain anatomy of professional and naïve car drivers we identified, in the former group, various areas characterized by increased gray matter density. These regions included basal ganglia, sensory-motor cortex, inferior frontal gyrus, retrosplenial cortex, fusiform/lingual gyrus and parahippocampus. A joint analysis between structural and functional results revealed that some of these areas—such as the bilateral posterior cingulate and retrosplenial cortex, left parahippocampus, left thalamus, left caudate nucleus, right lentiform nucleus, and right inferior frontal gyrus -, also showed a stronger degree of activation in professional as compared to naïve drivers during the passive-driving task. Interestingly, these structures have been associated with different aspects that may be relevant for driving behavior. In particular, the basal ganglia and the inferior frontal gyrus are known to be involved in execution and control of motor acts (Aron et al., 2004; Forstmann et al., 2008; Arsalidou et al., 2013), and their modification may indicate the acquisition of a specific motor repertoire and/or an increased ability in attaining accurate and timely motor reactions. On the other hand, other areas, including the retrosplenial and posterior cingulate cortex, and the parahippocampal gyrus, have been associated with spatial memory and navigation (Epstein, 2008; Shipman and Astur, 2008). Moreover, we also found the gray matter volume of the retrosplenial cortex to be specifically correlated with the level of proficiency in racing competitions, as expressed by the number of podia obtained with respect to the total number of races. This brain area, together with the hippocampus, is particularly important for the generation and retrieval of observer-independent spatial maps (i.e., allocentric spatial referencing) (Burgess, 2008; Vann et al., 2009; Galati et al., 2010; Wolbers and Hegarty, 2010; Sulpizio et al., 2013). In fact, several studies in both animal models (Sutherland et al., 1988; Vann et al., 2003; Harker and Whishaw, 2004) and human patients with lesions of the retrosplenial cortex (Takahashi et al., 1997; Ino et al., 2007) have demonstrated that this region is necessary for a correct spatial navigation through previously explored environments. Hence, it is tempting to speculate that the retrosplenial cortex might be involved in the storage of mental spatial maps of the racing tracks, and that the more prominent recruitment of this region in professional than in naïve drivers during the passive-driving task may depend on the retrieval of navigational information related to each circuit. This hypothesis is also consistent with a previous finding by Wolbers and Buchel (Wolbers and Buchel, 2005), who reported that retrosplenial activity during navigational learning in a virtual-reality town increased in parallel with the subject survey knowledge about the town. In this perspective, drivers characterized by a more developed retrosplenial cortex (either due to a genetic predisposition, or to plastic changes induced by years of practice or, more likely, to both) could be capable of obtaining the better spatial representation of a circuit and may be able to determine the optimal racing strategy. For instance, they could determine the best trait of the circuit for an overtaking, and thus may have more chances to win when competing against racers with a less efficient navigational representation.

### Methodological considerations

Functional MRI analyses described in the present work were based on data collected during a passive driving task in which subjects were presented with short movies depicting a Formula One car racing in different official circuits and imagined themselves driving the car. While an active task would resemble somehow more closely the actual driving competition environment, the use of a passive task offered important advantages, including the need of a much simpler technical equipment than a MRI compatible driving simulator, the possibility to minimize artifacts associated with movements in the scanner (Spiers and Maguire, 2007a) as well as to avoid potential confounding factors related to the different skill levels of the experimental groups (Poldrack, 2000). With regards to the latter issue, preliminary behavioral observations obtained using a Formula One driving simulator available at Formula Medicine in Viareggio, clearly indicated that naïve drivers experience major difficulties in driving a racing-car even in a simulated environment, making it impossible to use an active task if not only after a very extensive training.

In spite of the described advantages, the use of a passive task may be considered as a potential limitation for the present study, as one can object that observed differences between the two groups could simply be due to differences in the levels of attention and/or emotional participation. In addition, the driving video-clips were recorded on Formula One official circuits that were relatively familiar for most of the included racing-car drivers. Although we have no data from our subjects to directly exclude the role of attention levels, emotional response or familiarity, an involvement of these factors in determining the brain functional results is unlikely. In fact, both groups paid a great attention during the passive-driving task, as shown by the strong activation in the visual cortical areas, that is known to be modulated by attention to the task (Martinez et al., 2001), and as confirmed by the debriefing of participants upon completion of the study. Moreover, a previous study that evaluated the role of familiarity with a particular route in modulating cerebral activations during a passive-driving task demonstrated that brain response was higher in the sample with no direct experience on the specific track (Mader et al., 2009). The authors suggested that this may depend on a reduction in the levels of effort and attention needed to drive on known routes in more expert individuals. In this perspective, their findings support the exclusion of a major role of the attention level in determining results described in the present work. However, it will be important for future studies to specifically evaluate the potential contribution of familiarity in determining some of the differences between racing-car drivers and naïve drivers, for instance by including a control condition based on video-clips depicting a car running on non-official circuits. On the same line, the additional inclusion of standardized neuropsychological tests could have helped excluding the influence of other potential confounding factors that were not explored in the present study, such as general intelligence or level of education.

Finally, while the number of subjects included in the present work may appear to be relatively limited in light of the current standards for fMRI experiments and structural analyses (Friston, 2012), it should be kept in mind the exceptionality of the athletes sample, as the number of professional racing-car drivers with experience in Formula One, or other top level championships, is very limited to begin with, and comprises individuals who spend most of their time around the world, so that the recruitment of individuals who agree to travel to a research center to undergo testing—including MRI scan exams—is quite challenging. Furthermore, we posed quite restrictive inclusion criteria, so that subjects with any history of head trauma or accident or any other relevant medical condition would not be eligible for the study. It should be also emphasized that the consistency of results obtained using different analysis approaches, and the agreement with findings described by previous studies, including our own previous report (Bernardi et al., 2013), support the reliability of described functional and structural differences, despite the relatively limited number of participants.

### Conclusions

The present study demonstrated that during a passive racing-car driving task, professional and naïve drivers are characterized by different patterns of brain response, with a significantly greater involvement of areas devoted to motor control and visuo-spatial navigation in the first group and a prevalent focus on visual cortex in the naïve individuals. These results indicate that exceptional driving abilities may require the acquisition of a specific behavioral and functional motor repertoire that is different from the one associated with common “every day driving.” In addition, professional drivers revealed a significantly increased gray matter density in brain areas involved in motor planning/execution and spatial navigation, which have a clear potential relevance for a high-speed driving behavior. Of particular interest, among areas showing concomitant functional and anatomical changes, the retrosplenial cortex also showed a significant correlation with the driving proficiency in professional drivers. Finally, from a wider methodological perspective, these results support the reliability of approaches based on ISC analysis in studying driving behavior or other complex human behaviors. Indeed, this data driven method may be extremely useful when complex naturalistic stimuli are involved, as in the case of video-clips depicting sport-related activities.

In summary, in line with our previous observations (Bernardi et al., 2013), the present results indicate that although both professional and naïve drivers share the basic knowledge and experience needed for a common driving behavior, the specific expertise attained with high-speed racing-cars is associated with distinctive functional and structural correlates that sustain exceptional driving skills. To what extent such distinctive features in the brain morphological and functional architecture are a consequence of the intensive training undergone by professional drivers or rather precede and favor the achievement of their exceptional skills, cannot be resolved by cross-sectional studies. Brain studies in larger populations before and after intensive training will likely shed some light on this *nature vs. nurture* specific question.

### Conflict of interest statement

Formula Medicine is a profit organization that provides training-facilities and medical assistance for professional drivers. Riccardo Ceccarelli was paid employees of Formula Medicine at the time of data acquisition, and was directly involved in car racers recruitment and preliminary screening. No financial funding was received from Formula Medicine for this study.
