# Supplementary material for: It's not all in your car: functional and structural correlates of exceptional driving skills in professional racers
Source: Front Hum Neurosci. 2014 Nov 11;8:888. doi: 10.3389/fnhum.2014.00888 (PMC4227572; doi:10.3389/fnhum.2014.00888)
Supplement: Supplementary file 1 [file Table1.DOCX]

| **Brain Areas** | **Right Hemisphere** | | | **Left Hemisphere** | | |
| --- | --- | --- | --- | --- | --- | --- |
| **Prof. > Naïve** | **x** | **y** | **z** | **x** | **y** | **z** |
| Posterior Cingulate | 19 | -55 | 10 | -9 | -47 | 12 |
| Cingulate Cortex | 15 | -1 | 28 | -11 | -45 | 44 |
|  | 19 | -35 | 24 |  |  |  |
| Anterior Cingulate | - | - | - | -7 | 31 | -10 |
| Superior Frontal Gyrus | 23 | 53 | 12 | - | - | - |
| Middle Frontal Gyrus | 37 | 21 | 28 | -35 | 17 | 38 |
| Inferior Frontal Gyrus | 43 | 33 | 0 | -25 | 23 | -2 |
|  |  |  |  | -45 | 23 | 4 |
| Precuneus | 29 | -73 | 38 | -31 | -75 | 30 |
|  |  |  |  | -9 | -71 | 30 |
| Supramarginal Gyrus | 57 | 45 | 36 | -55 | -39 | 32 |
| Parahippocampal Gyrus | 45 | -19 | -16 | -31 | -31 | -10 |
|  |  |  |  | -13 | -9 | -12 |
| Middle Temporal Gyrus | 55 | -51 | 0 | -49 | -55 | 4 |
|  |  |  |  | -57 | -35 | 0 |
| Precentral Gyrus | 25 | -15 | 50 | - | - | - |
| Medial Frontal Gyrus | - | - | - | -17 | -9 | 50 |
| Caudate | 17 | 13 | 10 | -7 | 11 | 2 |
| Lentiform Nucleus | 13 | 3 | -4 | - | - | - |
| Thalamus | - | - | - | -3 | -11 | 8 |
| Cerebellum | 35 | -51 | 24 | - | - | - |
| **Naïve>Prof.** | x | y | z | x | y | z |
| Middle Occipital Gyrus | - | - | - | -35 | -77 | -8 |

**Supplementary Table S1**. Talairach coordinates for the centers of mass of voxel clusters that showed significantly different activation in the two groups during the passive driving task (*p*<0.01, FDR corrected).
