# Supplementary material for: It's not all in your car: functional and structural correlates of exceptional driving skills in professional racers
Source: Front Hum Neurosci. 2014 Nov 11;8:888. doi: 10.3389/fnhum.2014.00888 (PMC4227572; doi:10.3389/fnhum.2014.00888)
Supplement: Supplementary file 2 [file Table2.DOCX]

|  |  | **Occipital C.** | | **Parietal C.** | | **Temporal C.** | | **Insular C.** | | **Frontal C.** | | **Limbic C.** | | **Subcortical** | |
| --- | --- | --- | --- | --- | --- | --- | --- | --- | --- | --- | --- | --- | --- | --- | --- |
|  |  | Prof. | Naive | Prof. | Naive | Prof. | Naive | Prof. | Naive | Prof. | Naive | Prof. | Naïve | Prof. | Naive |
| L | Calcarine C. | - | 3 | - | - | - | - | - | - | 7 | - | - | - | - | - |
| R | Calcarine C. | - | 2 | - | 1 | - | - | - | - | 6 | - | - | - | - | - |
| L | Cuneus | - | 3 | - | - | - | 1 | - | 2 | 5 | 1 | - | - | - | - |
| R | Cuneus | - | 1 | - | - | - | 1 | - | - | 9 | - | - | - | - | - |
| L | Lingual G. | - | 1 | - | - | - | - | - | - | 2 | - | 2 | - | - | - |
| R | Lingual G. | - | 2 | - | - | - | - | - | - | 1 | - | 1 | - | - | - |
| L | Superior Occipital G. | - | 1 | 2 | 2 | - | - | - | - | 5 | 1 | 3 | - | - | - |
| R | Superior Occipital G. | - | - | - | 1 | - | - | - | - | 7 | - | 2 | - | - | - |
| L | Middle Occipital G. | - | - | - | 1 | - | - | - | - | 7 | - | 4 | - | - | - |
| R | Middle Occipital G. | - | - | 1 | 2 | - | - | - | - | 9 | - | 4 | - | - | - |
| L | Inferior Occipital G. | - | 1 | - | 2 | - | - | - | - | 6 | - | 4 | - | - | - |
| R | Inferior Occipital G. | - | - | - | - | - | - | - | - | 7 | - | 1 | - | - | - |
| L | Fusiform G. | - | - | - | - | - | - | - | - | 1 | - | - | - | - | - |
| R | Fusiform G. | - | 4 | - | 6 | - | - | - | - | - | 3 | - | - | - | - |
| L | Postcentral G. | - | - | - | - | - | - | - | - | 4 | - | - | - | - | - |
| R | Postcentral G. | - | 1 | - | - | - | - | - | - | 4 | - | - | - | - | - |
| L | Superior Parietal L. | - | - | - | - | - | - | - | - | 5 | - | 4 | - | - | - |
| R | Superior Parietal L. | - | 4 | - | - | - | - | - | - | 8 | - | 1 | - | - | - |
| L | Inferior Parietal L. | - | - | - | - | 2 | - | 1 | - | 3 | - | 6 | - | 3 | - |
| R | Inferior Parietal L. | - | 6 | - | - | 3 | - | - | - | 4 | - | 2 | - | - | - |
| L | Supramarginal G. | - | - | 1 | - | - | 1 | - | - | 2 | - | - | - | - | - |
| R | Supramarginal G. | - | 1 | 1 | - | 1 | - | - | - | 2 | - | 1 | - | - | - |
| L | Angular G. | 2 | - | 2 | - | 4 | - | 1 | - | 6 | - | 3 | - | 4 | - |
| R | Angular G. | - | 1 | - | 1 | - | 1 | - | - | - | - | - | - | - | - |
| L | Precuneus | 1 | - | - | - | - | - | - | - | 4 | - | - | - | - | - |
| R | Precuneus | - | - | - | 1 | - | - | - | - | 6 | - | - | - | - | - |
| L | Paracentral L. | - | 1 | - | - | - | - | - | - | 4 | 1 | - | - | - | 2 |
| R | Paracentral L. | - | 1 | - | - | - | - | - | 1 | 6 | 2 | - | - | - | 3 |
| L | Heschls G. | - | - | 3 | - | - | - | - | - | 10 | - | - | - | - | - |
| R | Heschls G. | - | - | 3 | - | - | - | - | - | 1 | - | - | - | - | - |
| L | Superior Temporal G. | - | - | 1 | - | 1 | - | - | - | 5 | - | - | - | - | - |
| R | Superior Temporal G. | - | - | 3 | - | - | - | - | - | 1 | - | - | - | - | - |
| L | Temporal Pole | - | - | - | - | - | - | - | - | 1 | - | - | - | - | - |
| R | Temporal Pole | - | - | - | - | - | - | - | - | - | - | - | 1 | - | - |
| L | Middle Temporal G. | - | - | - | - | 1 | - | 1 | - | 6 | - | - | - | - | - |
| R | Middle Temporal G. | - | - | - | 1 | - | - | - | - | 2 | - | - | - | - | - |
| L | Medial Temporal G. | - | - | - | - | - | - | - | - | - | - | - | - | - | - |
| R | Medial Temporal G. | - | 2 | - | - | - | - | - | - | - | - | - | - | - | - |
| L | Inferior Temporal G. | - | - | - | - | - | - | - | - | 3 | - | - | - | - | - |
| R | Inferior Temporal G. | - | - | - | 1 | - | - | - | - | 3 | - | - | - | - | - |
| L | Insula | - | 1 | - | - | - | - | - | - | 4 | - | - | - | - | 2 |
| R | Insula | - | 1 | 2 | 1 | 1 | - | - | - | 8 | - | - | - | - | - |
| L | Precentral G. | - | - | - | - | - | - | - | - | 8 | - | 4 | - | - | - |
| R | Precentral G. | - | 1 | - | - | - | - | - | - | 10 | - | 1 | - | - | - |
| L | Superior Frontal G. | 7 | - | 2 | - | 1 | - | 1 | - | 14 | - | 3 | - | 2 | - |
| R | Superior Frontal G. | 1 | - | - | - | 1 | - | 1 | - | 16 | - | 2 | - | - | - |
| L | Superior Orbital G. | 7 | - | 3 | - | 4 | - | 2 | - | 13 | - | - | - | 2 | - |
| R | Superior Orbital G. | - | - | 1 | - | 2 | - | - | - | 3 | - | - | - | - | - |
| L | Middle Frontal G. | 1 | - | - | - | - | - | - | - | 6 | - | 3 | - | - | - |
| R | Middle Frontal G. | - | - | - | - | 1 | - | - | - | 5 | - | - | - | - | - |
| L | Middle Orbital G. | 10 | - | 9 | - | 3 | - | 2 | - | 15 | - | 4 | - | 1 | - |
| R | Middle Orbital G. | 8 | - | 4 | - | 1 | - | 1 | - | 11 | - | 2 | - | - | - |
| L | Inferior Fro. G. (p.Op.) | - | 1 | - | - | - | - | - | - | 4 | - | - | - | - | 1 |
| R | Inferior Fro. G. (p.Op.) | - | 2 | 1 | - | - | - | - | - | 8 | - | 2 | - | - | - |
| L | Inferior Fro. G. (p.Tr.) | - | - | - | - | - | - | - | - | 2 | - | 3 | - | - | - |
| R | Inferior Fro. G. (p.Tr.) | - | - | - | - | - | - | - | - | 7 | - | 2 | - | - | - |
| L | Inferior Fro. G. (p.Or.) | - | - | - | - | - | - | - | - | 1 | - | - | - | - | - |
| R | Inferior Fro. G. (p.Or.) | - | - | - | - | - | - | - | - | 9 | - | 1 | - | - | - |
| L | Rolandic Operculum | - | 1 | 1 | 1 | 1 | - | - | - | 6 | - | - | - | - | - |
| R | Rolandic Operculum | - | - | 1 | 2 | - | - | - | - | 2 | - | - | - | - | - |
| L | Supplem. Motor A. | - | - | 1 | - | - | - | - | - | 8 | - | - | - | - | - |
| R | Supplem. Motor A. | - | - | - | - | - | - | - | - | 7 | - | - | - | - | - |
| L | Olfactory C. | - | - | - | - | 1 | - | - | - | - | - | - | - | - | - |
| R | Olfactory C. | 1 | - | - | - | 2 | - | - | - | - | - | - | - | 3 | - |
| L | Superior Med. Fro. G. | 10 | - | 10 | - | 4 | - | 1 | - | 18 | - | 3 | - | 6 | - |
| R | Superior Med. Fro. G. | 11 | - | 11 | - | 2 | - | - | - | 17 | - | 2 | - | 4 | - |
| L | Mid Orbital G. | 11 | - | 11 | - | 7 | - | 2 | - | 15 | - | 2 | - | 2 | - |
| R | Mid Orbital G. | 5 | - | 1 | - | - | - | - | - | 3 | - | - | - | - | - |
| L | Rectal G. | - | - | 2 | - | 2 | - | 2 | - | 3 | - | 4 | - | - | - |
| R | Rectal G. | - | - | - | - | - | - | - | - | 1 | - | 1 | - | - | - |
| L | Anterior Cingulate C. | 5 | - | 5 | - | - | - | - | - | 5 | - | - | - | - | - |
| R | Anterior Cingulate C. | 3 | - | 3 | - | - | 1 | - | - | 6 | - | - | - | - | - |
| L | Middle Cingulate C. | - | - | 2 | - | - | - | - | - | 8 | - | - | - | - | 1 |
| R | Middle Cingulate C. | - | - | 2 | - | - | - | - | - | 8 | - | - | - | - | - |
| L | Posterior Cingulate C. | 8 | - | 2 | - | - | - | - | - | 5 | - | - | - | 3 | - |
| R | Posterior Cingulate C. | 5 | - | 3 | - | - | - | - | - | 2 | - | - | - | - | - |
| L | Hippocampus | - | - | - | - | - | - | - | - | - | - | 2 | - | 2 | - |
| R | Hippocampus | - | - | - | - | - | - | - | - | - | - | 3 | - | - | - |
| L | Parahippocampus | - | - | - | - | - | - | - | - | 1 | - | - | - | - | - |
| R | Parahippocampus | - | - | - | - | - | - | - | - | 2 | - | 3 | - | 8 | - |
| L | Amygdala | - | - | - | - | - | - | - | - | 1 | - | 1 | - | - | - |
| R | Amygdala | - | - | - | - | - | - | - | - | 1 | - | 3 | - | 1 | - |
| L | Caudate | - | - | - | - | - | - | - | - | 1 | - | 1 | - | - | - |
| R | Caudate | - | - | 2 | - | - | - | - | 1 | 2 | - | 1 | - | - | 1 |
| L | Putamen | - | - | 1 | 1 | - | - | - | - | 3 | - | 1 | - | - | 1 |
| R | Putamen | - | - | 2 | - | - | - | - | - | 6 | - | 2 | - | - | - |
| L | Pallidum | - | - | 1 | - | - | - | - | - | - | - | 2 | - | - | - |
| R | Pallidum | - | - | 1 | 1 | - | - | - | - | 1 | - | 3 | - | - | - |
| L | Thalamus | - | - | - | 1 | - | - | - | 1 | 3 | 1 | 2 | 1 | - | - |
| R | Thalamus | - | - | - | 2 | - | - | - | - | 4 | - | 2 | - | - | - |

**Supplementary Table S2**. For each of the 90 brain regions of interest (ROI) used in the functional connectivity analysis the table shows, for professional and naïve drivers, the number of significantly stronger connections (*p*<0.05) with other ROIs belonging to different brain ‘macro-areas’ including occipital, parietal, temporal, insular, frontal, and limbic cortex, and subcortical structures. This subdivision is the same used in Figure 3.
