# Supplementary material for: It's not all in your car: functional and structural correlates of exceptional driving skills in professional racers
Source: Front Hum Neurosci. 2014 Nov 11;8:888. doi: 10.3389/fnhum.2014.00888 (PMC4227572; doi:10.3389/fnhum.2014.00888)
Supplement: Supplementary file 3 [file Table3.DOCX]

| **Brain Areas** | **Right Hemisphere** | | | **Left Hemisphere** | | |
| --- | --- | --- | --- | --- | --- | --- |
| **Prof. > Naïve** | **x** | **y** | **z** | **x** | **y** | **z** |
| Thalamus | 3 | -17 | 4 | -3 | -17 | 4 |
| Lentiform Nucleus | 25 | 5 | -8 | -17 | 5 | -8 |
| Fusiform Gyrus | 45 | -39 | -26 | -45 | -31 | -20 |
| Inferior Frontal Gyrus | 41 | 17 | 0 | -43 | 23 | -4 |
| Precentral Gyrus | 42 | 11 | 10 | -51 | 3 | 16 |
| Lingual Gyrus | - | - | - | -12 | -62 | -1 |
| Insula | - | - | - | -45 | -17 | 8 |
| Postcentral Gyrus | - | - | - | -55 | -13 | 16 |
| Parahippocampal Gyrus | - | - | - | -33 | -23 | -22 |
| Posterior Cingulate (RSC) | 5 | -61 | 10 | -9 | -63 | 10 |
| Medial Frontal Gyrus | 9 | 57 | 11 | - | - | - |
| Declive | - | - | - | -3 | -64 | -19 |
| Culmen | 1 | -57 | -3 | - | - | - |

**Supplementary Table S3**. Talairach coordinates for the centers of mass of voxel clusters that showed significantly different gray matter density in the two groups (p<0.05 TFCE cluster corrected). RSC: RetroSplenial Cortex.
